# The Receptor Slamf1 on the Surface of Myeloid Lineage Cells Controls Susceptibility to Infection by *Trypanosoma cruzi*

**DOI:** 10.1371/journal.ppat.1002799

**Published:** 2012-07-12

**Authors:** Jossela Calderón, Elena Maganto-Garcia, Carmen Punzón, Javier Carrión, Cox Terhorst, Manuel Fresno

**Affiliations:** 1 Centro de Biología Molecular “Severo Ochoa” (CSIC-UAM), Universidad Autónoma de Madrid, Cantoblanco, Madrid, Spain; 2 Instituto de Investigación Sanitaria Princesa (IP), Madrid, Spain; 3 The Division of Immunology, Beth Israel Deaconess Medical Center, Harvard Medical School, Boston, Massachusetts, United States of America; National Institute of Health, United States of America

## Abstract

*Trypanosoma cruzi*, the protozoan parasite responsible for Chagas' disease, causes severe myocarditis often resulting in death. Here, we report that *Slamf1*−/− mice, which lack the hematopoietic cell surface receptor Slamf1, are completely protected from an acute lethal parasite challenge. Cardiac damage was reduced in Slamf1−/− mice compared to wild type mice, infected with the same doses of parasites, as a result of a decrease of the number of parasites in the heart even the parasitemia was only marginally less. Both *in vivo* and *in vitro* experiments reveal that Slamf1-defIcient myeloid cells are impaired in their ability to replicate the parasite and show altered production of cytokines. Importantly, IFN-γ production in the heart of Slamf1 deficient mice was much lower than in the heart of wt mice even though the number of infiltrating dendritic cells, macrophages, CD4 and CD8 T lymphocytes were comparable. Administration of an anti-Slamf1 monoclonal antibody also reduced the number of parasites and IFN-γ in the heart. These observations not only explain the reduced susceptibility to *in vivo* infection by the parasite, but they also suggest human Slamf1 as a potential target for therapeutic target against *T. cruzi* infection.

## Author Summary

Chagas' disease caused by the intracellular protozoan *Trypanosoma cruzi* is the most important parasitic infection in Latin America affecting several million persons. Nonetheless, there is no therapy or vaccine available. Thus, more efforts are needed to identify new therapeutic targets. Here, we report that Slamf1, which controls phagosomal/lysosomal fusion and phagosomal NADPH-oxidase activity, is required for *T.cruzi* replication in macrophages and dendritic cells, but not in other cells, which do not express the receptor. In the absence of Slamf1 we detect reduced number of parasites in the heart compared to infected wt mice. This explains why *T. cruzi*-infected *Slamf1* deficient mice do not succumb to myocarditis induced by a lethal challenge with *T. cruzi* in contrast to BALB/c mice. Perhaps more importantly, we demonstrate that parasite replication in phagocytes is of far greater importance for the pathogenesis of the cardiomyopathy than replication in other cells. Moreover, we found much lower IFN-γ production in the heart of Slamf1 deficient mice than in the heart of BALB/c mice. We corroborated those results using an alternative approach, blocking Slamf1 function in vivo by treating mice with anti-Slamf1 antibodies. Consequently, Slamf1 is an attractive novel therapeutic target for modulating *T. cruzi* infection.

## Introduction

American trypanosomiasis (Chagas' disease) is caused by the intracellular protozoan *Trypanosoma cruzi* that is transmitted to vertebrate hosts by insect vectors belonging to the *Reduviidae* family [1]. It is one of the most important parasitic infection in Latin America affecting several million persons in South and Central America [2] Due to the immigration Chagas' disease is now considered an emergent one in Europe [3]. The disease is a complex zoonosis, with mammals as natural reservoir hosts. Transmission is primarily by contact with the contaminated faeces of domiciliated blood sucking triatomine bugs. The life cycle of this parasite alternates between three morphologically distinct forms: infective (metacyclic or blood trypomastigotes), insect borne (epimastigotes) which replicate in the vector and intracellular replicative (amastigotes) which grow and replicate intracellularly in a variety of mammalian cells, including macrophages, cardiomyocytes and muscle fibers [4]–[6].

Myocarditis is the most serious and frequent manifestation of acute and chronic infection [2]. The pathogenesis is thought to be triggered by parasites in the lesions and dependent on an immune-inflammatory response to them [7]–[9]. Activation of a T helper type (Th_1_) response, that release IFN-γ and TNF, is required to activate the microbicidal activity of macrophages important in the control of *T. cruzi* infection [10], [11]. Nonetheless, the development of severe cardiomyopathy in Chagas' disease is also thought to be due to a Th_1_-specific immune response [12].

*T. cruzi* infects a variety of host cells, including macrophages and cardiomyocytes. Several *T. cruzi* molecules, glycoproteins, trans-sialidase and mucins among others, play a role in cell invasion mainly interacting with TLRs or mannose receptors [13]–[17].

The Signaling Lymphocytic Activation Molecule family (Slamf) receptors are adhesion molecules that are involved in signaling between immune cells regulating for instance T cell proliferation, antibody production, cytotoxic responses and cytokine production, *e.g.* IFNγ [18]–[25]. The self-ligand adhesion molecule Slamf1 (CD150) is not only a co-stimulatory molecule at the interface between antigen presenting cells and T cells, but also functions as a microbial sensor. For instance, Slamf1 also binds to the hemaglutinin of Measles virus and to an outer membrane protein of *E.coli* and *S.typhimurium* [26], [27]. The latter interaction drives Slamf1 into the *E.coli* phagosome where the receptor positively controls the microbicidal activity of macrophages by a signaling mechanism that is distinct from its signaling as an adhesion molecule [27]. Because Slamf1 partakes in bactericidal responses as the receptor and plays a role in protecting against infection with *Leishmania major* [23] we set out to evaluate how *Slamf1*-deficient mice would respond to an infection by the intracellular parasite *T. cruzi*. Surprisingly, we find that *Slamf1−/−* mice are resistant to a lethal dose of *T. cruzi*, because the number of parasites in the heart is greatly reduced as compared to infected *wt* mice. Further *in vivo* and *in vitro* experiments revealed that *T. cruzi* has impaired ability to replicate into Slamf1-deficient myeloid cells. Administration of an anti-Slamf1 monoclonal antibody also reduced the number of amastigotes in the heart.

## Results

### ***Slamf1^−/−^*** mice survive an acute lethal infection by *T. cruzi*

**Figure 1:**
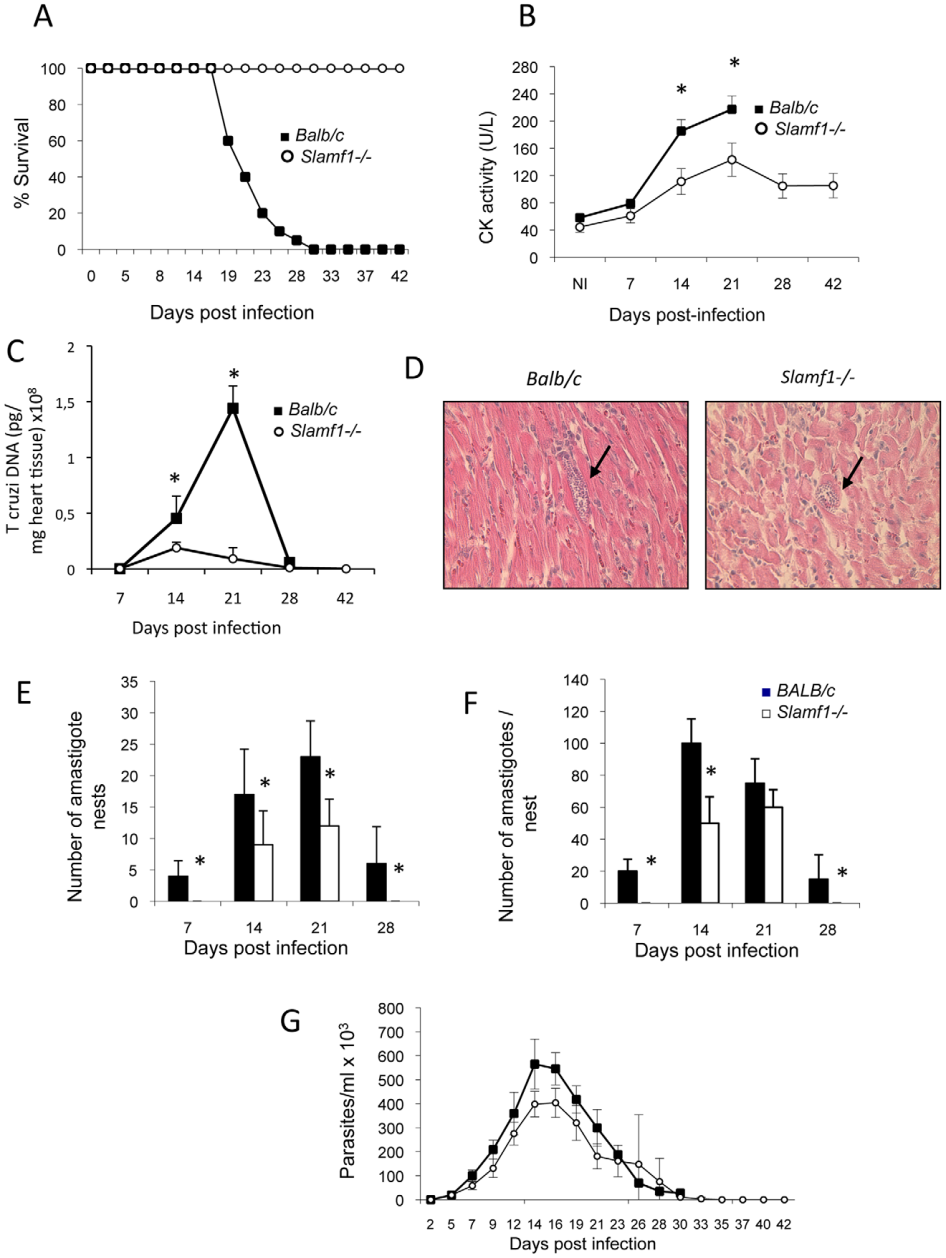
Time-course of *T. cruzi* infection in *Slamf1^−/−^* mice and reduced *T. cruzi* infection in the hearts from *Slamf1−/−* animals. BALB/c or *Slamf1^−/−^* mice were intraperitoneally infected with 2×10^3^ trypomastigotes of the *T. cruzi* Y strain and were sacrificed at different dpi. A) Survival. B) Serum CK levels. Analysis of *T. cruzi* presence in heart tissue from *T. cruzi* infected BALB/c or *Slamf1^−/−^* animals: C) Quantification of *T. cruzi* DNA in the heart tissue of infected BALB/c- and *Slamf1^−/−^* mice. *T. cruzi* DNA is expressed as the amount of parasite DNA obtained from a heart tissue sample (pg of parasite DNA/mg of heart tissue). Results are expressed as the mean values (±SD) for triplicates of pooled DNA from 5 different mice. A representative experiment of the 3 performed is shown. D) Histochemical analysis by Hematoxylin-Eosin stain. A representative field is shown. E) Quantification of the number of amastigote nests per 20 fields. F) Average number of amastigotes/nest per 20 fields. At least 20 fields were observed of each preparation (3 preparations/mouse and 3 mice per group). Results are expressed as the mean values (±SD) for 100 independent microscopic fields from 5 different mice (20 each). G) Blood parasitemia. (*) Statistically significant differences between *Slamf1^−/−^* mice and BALB/c (p>0.05).

*Slamf1^−/−^* and *Slamf1+/*+ *BALB/c* mice were infected with the highly virulent *T. cruzi* Y strain. Interestingly, unlike *wt* mice, *Slamf1^−/−^* mice did not die from the infection and eventually recovered (Figure 1A). This complete resistance was observed also with a very high parasite inoculum (10^4^ parasites/mouse) (data not shown). In the acute phase of the infection *T. cruzi* induces myocarditis, which is thought to be the ultimate cause of mortality [2], [9]. Indeed the creatinine kinase (CK) levels, a marker of cardiac damage, were significantly lower in the serum of infected *Slamf1^−/−^* mice than in *Slamf1*-sufficient BALB/c mice (Figure 1B), suggesting that reduced heart damage was the cause of the survival of the *Slamf1^−/−^* mice.

To assess the numbers of *T. cruzi* present in the heart of infected *wt* and mutant mice, quantitative QC-PCR with parasite specific probes was used. In *BALB/c* mice an increase in parasite load, which follows parasitemia, peaked at 21 days postinfection (dpi). By contrast, *Slamf1^−/−^* mice had a much smaller *T. cruzi* load in their hearts (Figure 1C). Next, we performed histological analysis of the infected hearts and compared the numbers of *T. cruzi* amastigotes present in the cardiomyocytes of infected *Slamf1^−/−^* and *Slamf1^+/+^ BALB/c* mice. First, amastigote nests were less frequently observed in the hearts of *Slamf1^−/−^* than of *Slamf1^+/+^* mice and appeared to be smaller in size (Figure 1D). Furthermore, the number of amastigote nests in *Slamf1^−/−^* mice from 7 dpi until 28 dpi was dramatically decreased compared to *wt BALB/c* mice (Figure 1E), although the kinetics were similar as the maximum number of amastigote nests was at 21 dpi, in close concordance with the parasite DNA levels. In addition, at all time points, the number of amastigotes per nest was lower in infected *Slamf1^−/−^* mice than in *Slamf1^+/+^ BALB/c* mice (Figure 1F). By contrast, the number of parasites in the blood followed similar kinetics in both mouse strains, although parasitemia was slightly lower than in *Slamf1−/−* mice than in *Slamf1^+/+^ BALB/c* mice (Figure 1G). Taken together, the data clearly demonstrate that infected *Slamf1^−/−^* mice have much less *T. cruzi* amastigotes in their hearts than *Slamf1^+/+^* littermates, which is the most likely cause for the survival of *Slamf1^−/−^* mice upon an acute infection by the parasite.

### Leukocyte responses in the heart of *Slamf1−/−* mice infected with *T.cruzi*

**Figure 2:**
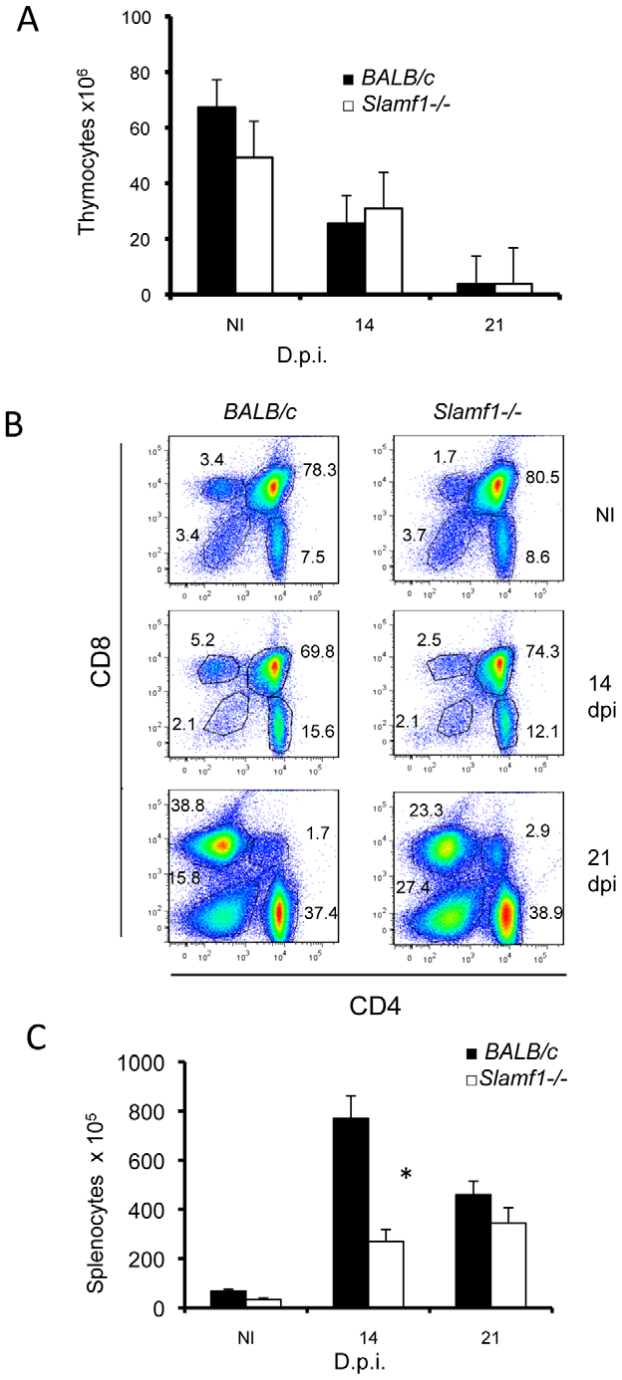
Immunologic populations from lymphoid organs of *T. cruzi*-infected mice. Thymocytes were isolated from thymus from control NI or *T. cruzi* infected BALB/c or *Slamf1^−/−^* mice at 14 and 21 dpi. A) Total number of thymocytes isolated from NI or infected mice. Results are expressed as the mean values (±SD) for triplicates and from 5 different mice. B) CD4 and CD8 thymocytes in infected mice. Thymocytes were analyzed by two-color flow cytometry. Numbers represent % of CD4, CD8 SP, DP or DN thymocytes. Thymocytes from 5 mice in each group were pooled and analyzed. C) Spleens were isolated from infected mice and the total number of lymphocytes/spleen was quantified. Results are expressed as the mean values (±SD) for triplicates and from 5 different mice. (*) Statistically significant differences between *Slamf1^−/−^* mice and BALB/c (p>0.05).

As reported previously [28], infection of *BALB/c* mice with *T. cruzi* abrogates thymocyte development with kinetics closely following the increased numbers of circulating parasites, as judged by the loss of thymocyte numbers (Figure 2A). A reduction of the CD4^+^ CD8^+^ double positive compartment was also observed (Figure 2B), in agreement with the findings by Perez et al [29], which could be caused by TNF, corticosteroids, parasite trans-sialidase, extracellular ATP, androgens or galectin-3 [28], [29]. However, in spite of the complete survival of the *Slamf1^−/−^* mice, the kinetics of depletion of thymocyte subpopulations was identical as that in *wt BALB/c* mice (Figure 2B). During infection of *BALB/c* mice with *T. cruzi* splenic cellularity increased approximately 8-fold (Figure 2C) By contrast, this expansion was only 3-fold in the spleens from *Slamf1^−/−^* mice indicative of a much lesser activation state at early times although at day 21 d.p.i. cellularity increases were similar. Moreover, the distribution of leukocyte subpopulations in spleens was similar in the *Slamf1^−/−^* and *wt* mice (Figure S1). Taken together, the data indicate that the difference in susceptibility of *Slamf1−/−* and wt *BALB/c* mice could not be attributed to selective differences in the alterations of major leukocyte subpopulations in those organs affected by the infection.

**Figure 3:**
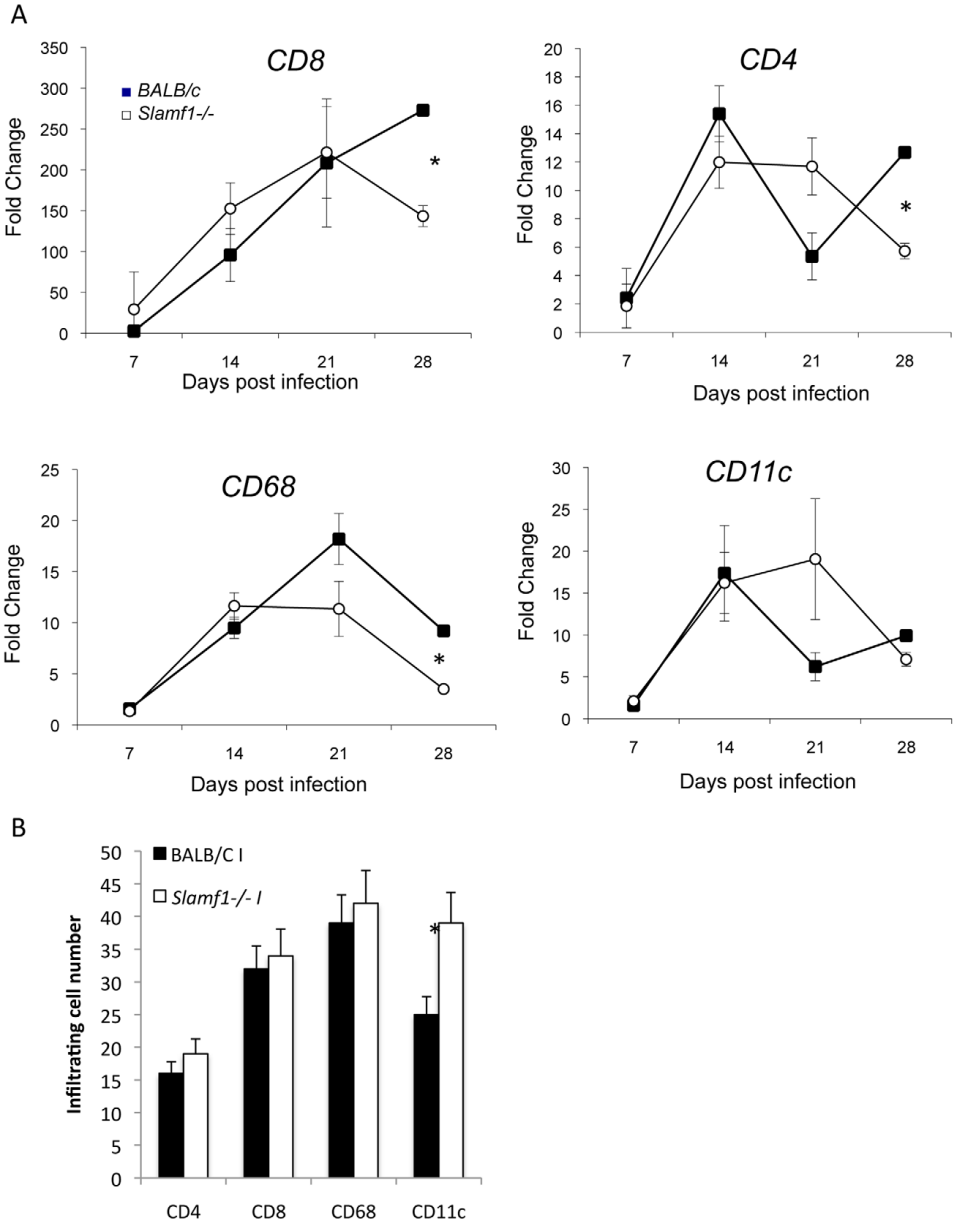
Heart leukocyte infiltration during *T. cruzi* infection. Cell populations in mouse heart tissue during *T. cruzi* infection. A) Cell subset infiltration quantified by QC-PCR. Total RNA was isolated in heart tissue obtained from mice at different dpi, and quantitative reverse-transcriptase polymerase chain reaction was performed as described in Materials and Methods. Results are expressed as the logarithm of relative quantity (RQ) calculated from comparative threshold cycle values, as described in Material and Methods. mRNAs values are shown for DCs (CD11c), CD4 and CD8 T lymphocytes and macrophages (CD68). Results are expressed as the mean values (±SD) for triplicates of pooled DNA from 3 different mice. A representative experiment of the 3 performed is shown. B) Evaluation of infiltrating subpopulations by confocal analysis. Hearts were fixed and stained with anti-CD4, CD8, CD68 and CD11 as described in Methods. Results shown are the mean number of cells (±SD) per 10 fields (20 independent microscopic fields from 3 different mice were counted). (*) Statistically significant differences between *Slamf1^−/−^* mice BALB/c (p>0.05).

One of the key contributors to the cardiomyopathy during infection with *T. cruzi* is thought to be the infiltration by CD8 T lymphocytes, which as a consequence of a immune reactivity to the parasite produce an inflammatory milieu that is detrimental for heart function [8]. We therefore determined CD8 infiltration into the heart of infected mice by mRNA levels of subpopulation-specific cell surface markers [30]. CD8 infiltration increases constantly into the hearts of *BALB/c* mice until their death (Figure 3A). However, no significant differences were observed in the kinetic of CD8 infiltration (7 to 21 dpi) between *Slamf1−/−* and *BALB/c* mice except for a small decrease in CD8 T cell infiltration at 28 dpi, likely reflecting the resolution of infection in this strain of mice (Figure 3). Similarly, no significant differences were observed in the kinetics of CD4 T lymphocyte infiltration (Figure 3A). Uninfected hearts have no detectable mRNA of those markers.

We evaluate whether myeloid cells infiltration into the heart was different between *T.cruzi*-infected mutant and *wt* mice using a similar approach. As previously shown [30], macrophage (CD68) and dendritic cell (CD11c) infiltration into the heart of wt mice infected with the parasite peaks at 21 days post-infections (Figure 3A). However, only statistically significant modest decrease in CD68^+^ macrophage infiltration at later times after infection (28 dpi) into the hearts of infected *Slamf1−/−* compared to *BALB/c* mice were observed. Those results were confirmed using confocal microscopy of the infected hearts with specific antibodies. No statistically significant differences in CD4, CD8 or CD68 infiltration and a slight increase in CD11c were observed at 21 dpi (Figure 3B).

Taken together, these data indicate that the striking difference in susceptibility of Slamf1−/− and wt BALB/c mice could not be attributed to differences in the recruitment of effector CD8 or CD4 T cells or myeloid cells into the heart.

### Deviation of cytokine responses in the heart of *Slamf1−/−* mice infected with *T. cruzi*

**Figure 4:**
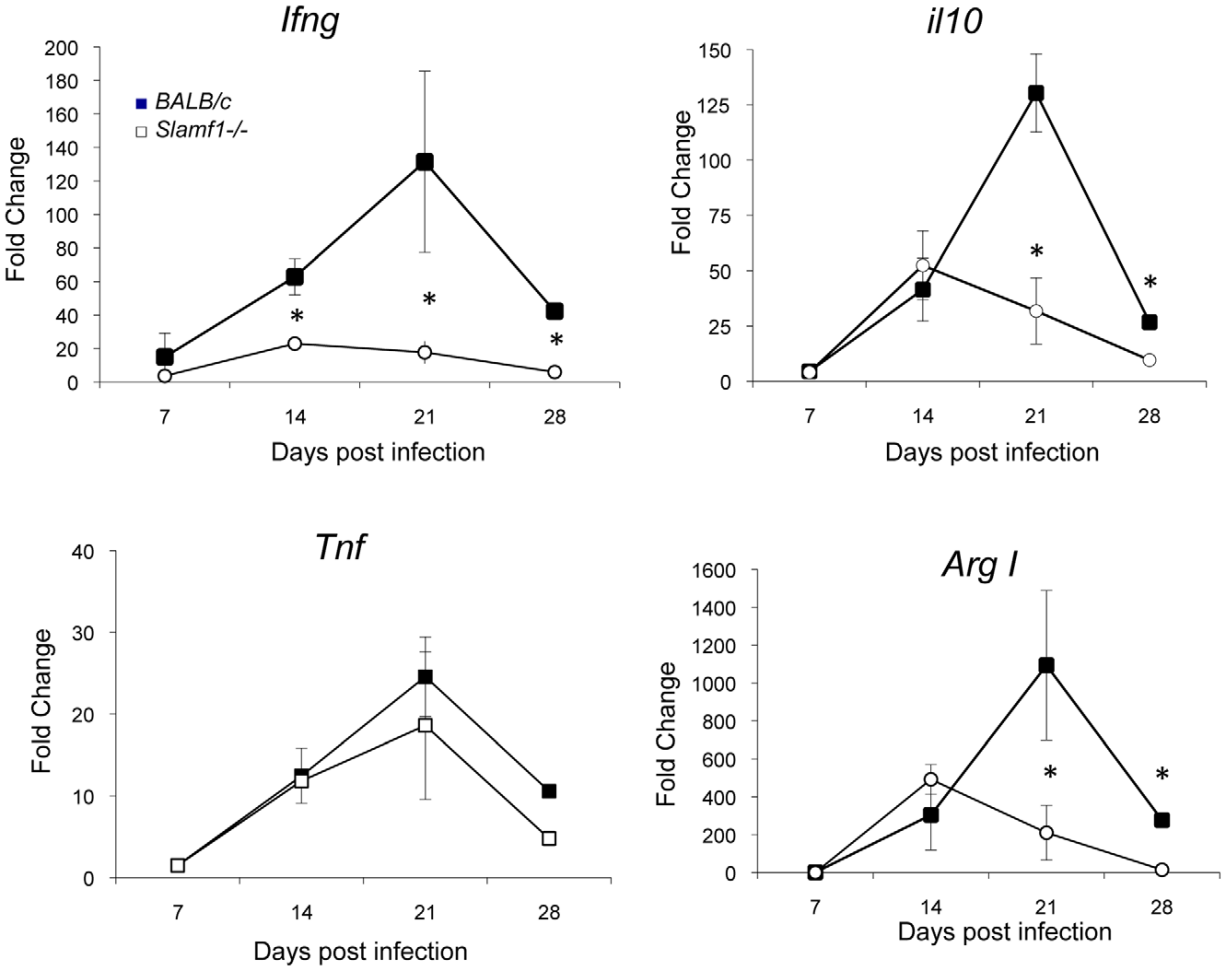
Heart cytokine and immune modulator production by *T. cruzi* infected mice. Cytokine mRNA production in the heart of *T. cruzi* infected mice was evaluated by QC-PCR as described in Methods. Total RNA was isolated in heart tissue obtained from BALB/c and *Slamf1^−/−^* mice at different dpi, and quantitative reverse-transcriptase polymerase chain reaction was performed as described in Materials and Methods. Results are expressed as the logarithm of relative quantity (RQ) calculated from comparative threshold cycle values, as described in Material and Methods. (*) Statistically significant differences between *Slamf1^−/−^* mice and BALB/c (p>0.05).

We next tested the levels of cytokines in the hearts by QC-PCR. In *BALB/c* mice, *T. cruzi* infection is accompanied by an increase in the heart of pro-inflammatory mediators TNF, IL-6 and IFNγ as well as anti-inflammatory mediators, *e.g.* IL-10 and arginase I [30], [31]. However, a significant reduction in IFNγ and IL-10 mRNA in the hearts of infected *Slamf1−/−* mice, as compared to *BALB/c*, was observed (Figure 4), TGF-β, IL-4 and IL-13, TNFα and IL-6 mRNA levels were no different to those in *wt* mice (Figure 4 and Figure S2). Interestingly, lower levels of arginase I mRNAs were also observed in *Slamf1−/−* mice especially at the peak of parasite load 21 dpi (Figure 4C), consistent with our previous finding that sustained arginase I expression through the acute infection is detrimental for the host [31].

In contrast, systemic cytokine production detected in the sera of infected mice was not different (Figure S3), once again indicating again that changes in systemic immune responses are not responsible for the reduced susceptibility of *Slamf1−/−* mice to *T. cruzi* infection.

### Reduced replication of *T. cruzi* in *Slamf1−/−* macrophages

**Figure 5:**
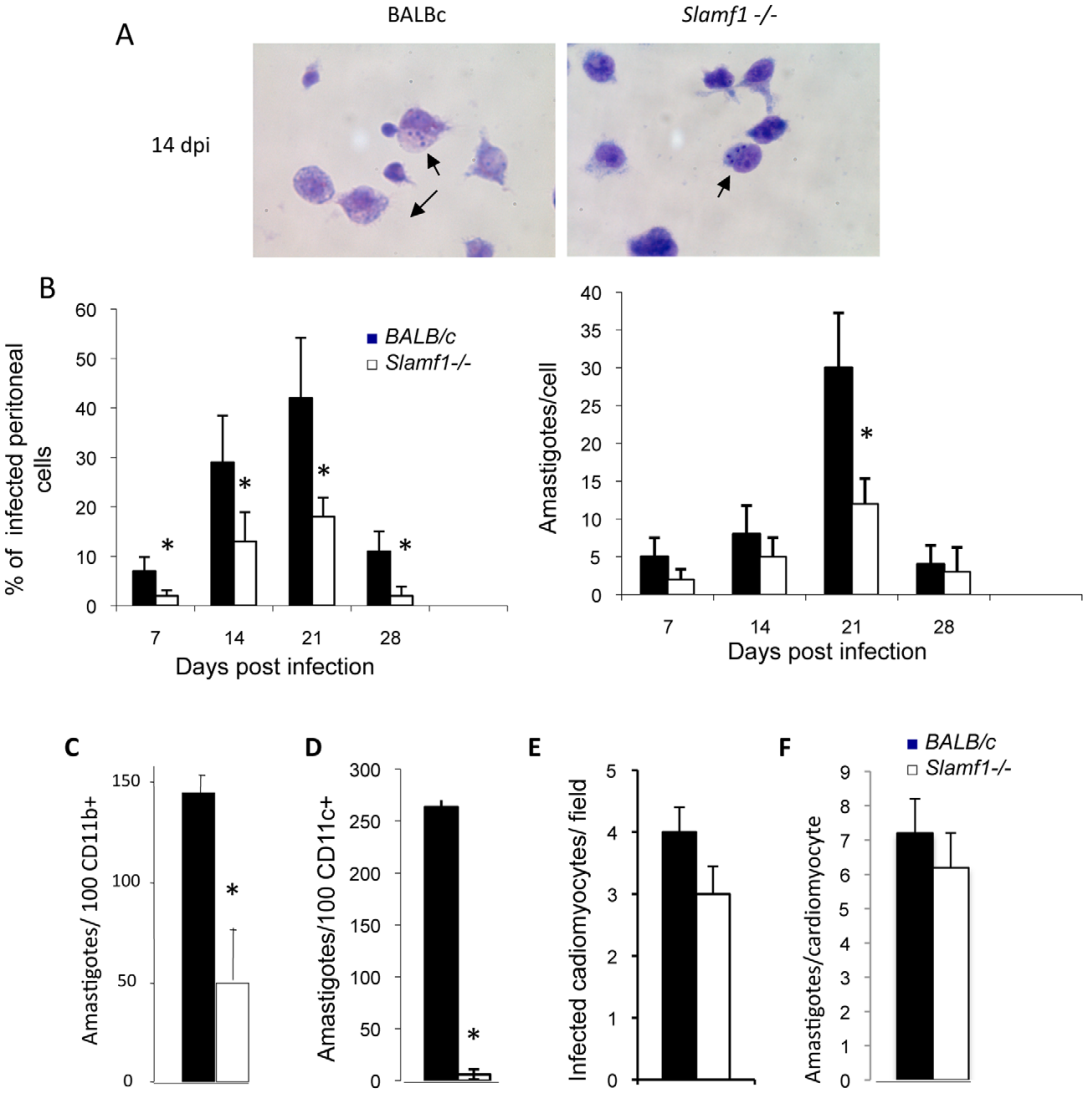
Slamf1 deficient myeloid cells are less susceptible to *T. cruzi* infection. Mice were intraperitoneally infected with *T. cruzi* and at 0, 7, 14, 21 and 28 dpi mice were sacrificed. A) Giemsa staining of adherent peritoneal macrophages from *Slamf1* ^−/−^ and BALB/c animals were isolated by intraperitoneal lavage with PBS and stained. A representative field is shown. B) Quantification of infected adherent cells in the peritoneal lavages. Quantification of the number of amastigotes nests per 20 field and average number of amastigotes/nest per 20 fields. Results are expressed as the mean values (±SD) for 100 independent microscopically fields from 5 different mice (20 each). C) Peritoneal macrophages and D) DC cells from BALB/c and *Slamf1^−/−^* mice were infected in vitro with *T. cruzi* (10 parasites/cell). The number of amastigotes released to the supernatant after 48 of infection was estimated by counting them by optical microscopy. Results are expressed as the mean values (±SD) for triplicates from 3 different experiments. E and F) Neonatal cardiomyocytes were infected “in vitro” with *T. cruzi* and 72 h postinfection analyzed by Giemsa staining. Quantification of the number of infected cardiomyocytes per field (E) and average number of amastigotes/cardiomyocyte (F). Results are expressed as the mean values (±SD) for 100 independent microscopic fields from 5 different mice (*) Statistically significant differences between *Slamf1^−/−^* mice and BALB/c (p>0.05).

An additional, and perhaps more relevant, explanation for the reduced amastigote content in the hearts of *Slamf1−/−* mice, could be that intracellular *T. cruzi* replication was impaired in the mutant mice. To address this hypothesis, we analyzed macrophages isolated from the peritoneum of mice that were infected with *T. cruzi*. Adherent peritoneal cells from 7 dpi to 28 dpi of infected *BALB/c* mice contain intracellular amastigotes, with a number that peaked at 21 dpi when approximately 50% of the peritoneal macrophages from *BALB/c* mice are infected by *T. cruzi* (Figure 5A and B). Those infected cells at 21 dpi contained 30 amastigotes. On average, by contrast, a maximum of only 20% of adherent cells from the peritoneal fluid of *Slamf1−*/− mice borne the parasite, with a maximum of 15 amastigotes/cell at 21 dpi (Figure 5B). Combining those 2 parameters, the reduction in amastigote content in peritoneal cells isolated from *Slamf1−*/− mice was greater than 75% as compared with wt mice. Thus, peritoneal macrophages isolated from *T.cruzi* infected *Slamf1−*/− mice appeared to replicate the parasite less efficiently.

To corroborate this observation, we tested whether *in vitro T. cruzi* infection was impaired in isolated *Slamf1−*/− macrophages, dendritic cells (DC) or cardiomyocytes. Forty-eight hours after infection replication of the parasite and the generation of amastigotes was detected in *wt* cells (Figure 5C). However, *Slamf1−*/− macrophages (Figure 5C) and DCs (Figure 5D) were far less effective in supporting *T. cruzi* replication then *wt* cells. In contrast to myeloid cells, *Slamf1−*/− and wt cardiomyocytes were equally susceptible to *in vitro* infection with *T. cruzi* (Figure 5E and F).

**Figure 6:**
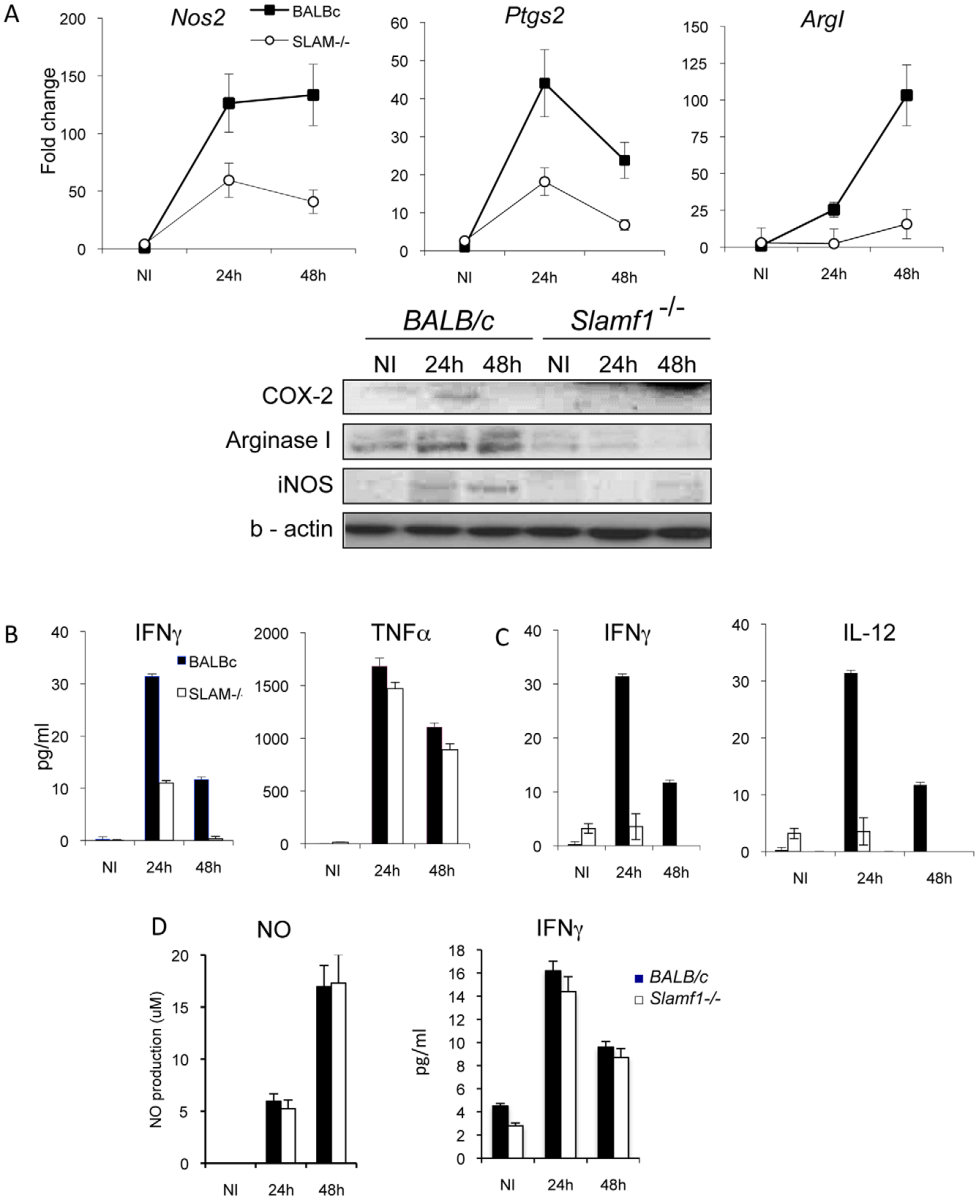
Cytokine production and immune modulators by “in vitro” *T. cruzi* infected DC, macrophages or cardiomyocytes. Peritoneal macrophages, DC or cardiomyocytes from *Slamf1^−/−^* from BALB/c mice were infected in vitro with *T. cruzi*. A) Cox-2, iNOS and Arginase mRNA, evaluated by QC-PCR production (upper graphs) and protein by western blot (lower gels) by infected macrophages at 24 or 48 hr post infection as described in Methods. B) Cytokine (IFN-γ and TNF) release to supernatants from infected macrophages was evaluated by ELISA 24 or 48 hr post infection as described in Methods. C) Cytokine (IFN-γ and IL-12) release to supernatants from infected DCs was evaluated by ELISA 24 or 48 hr post infection as described in Methods. D) IFN-γ and NO production by infected cardiomyocytes. NO was evaluated by Gris reaction and IFN-γ by ELISA. Results are expressed as the mean values (±SD) for triplicates from 3 different experiments. (*) Statistically significant differences between *Slamf1^−/−^* and BALB/c cells (p>0.05).

Next, we analyzed whether Slamf1 deficiency alters myeloid cell response to the parasite. As expected, *T. cruzi* infection of *wt* macrophages triggered the production of inflammatory mediators inducible nitric oxide synthase (iNOS *Nos2*) and cyclooxygenase-2 (COX-2, *Ptgs2*) at the mRNA and protein level (Figure 6A). Also arginase 1, *Arg1*, mRNA was induced upon infection. In contrast, *T. cruzi* infection of *Slamf1−/−* macrophages and show reduced levels of *Ptgs2, Nos2 and Arg1* mRNAs. Moreover, infected *Slamf1−/−* macrophages release less IFNγ into the supernatant than infected BALB/c macrophages but similar levels of TNF (Figure 6B). Similarly, upon *T. cruzi* infection *Slamf1−/−* DC also produced less IFNγ and IL-12 than *wt* BALB/c DCs (Figure 6C). Not surprisingly, because cardiomyocytes do not express Slamf1, upon infection with *T. cruzi* these cells, whether isolated from *wt* or *Slamf1−/− BALB/c* mice, produced equal amounts of IFNγ and nitric oxide (NO) upon in vitro infection (Figure 6D). Taken together, the outcomes of these experiments support a model in which *T. cruzi* replication is reduced and cytokine production is altered when Slamf1 is absent in macrophages and DCs.

### A monoclonal antibody directed against Slamf1 reduces the number of *T. cruzi* amastigotes in the heart

**Figure 7:**
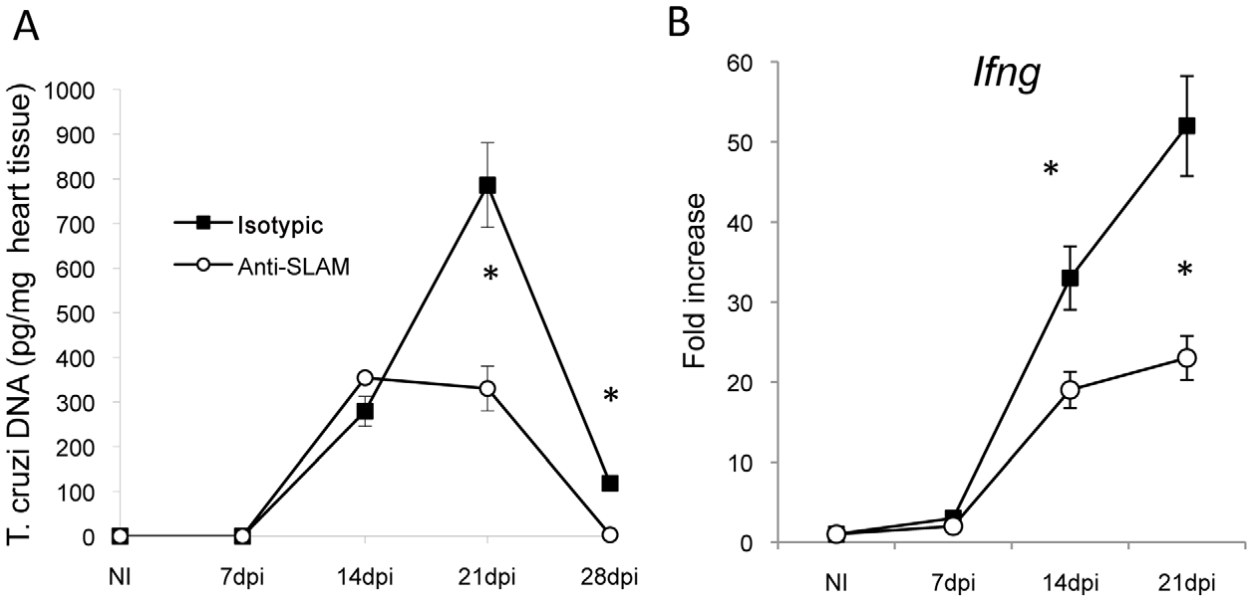
Anti-Slamf1 antibodies reduce heart parasite load. BALB/c or *Slamf1^−/−^* mice were intraperitoneally infected with 1×10^2^ trypomastigotes of the *T. cruzi* Y strain and treated with anti Slamf1 or control antibodies (0.5 mg/mouse once a week). At different dpi mice were sacrificed. A) *T. cruzi* DNA was quantified in the heart tissue of infected mice and expressed as the number of picograms of parasite DNA per milligram of DNA obtained from a heart tissue sample. Results are expressed as the mean values (±SD) for triplicates of pooled DNA from 5 different mice. A representative experiment of the 2 performed is shown. B) IFN-γ mRNA production in the heart of *T. cruzi* infected mice. Total RNA was isolated in heart tissue at different dpi, and quantitative reverse-transcriptase polymerase chain reaction was performed as described in Materials and Methods. Results are expressed as the logarithm of relative quantity (RQ) calculated from comparative threshold cycle values, as described in Material and Methods. (*) Statistically significant differences between *Slamf1^−/−^* mice and BALB/c (p>0.05).

To test this concept we employed an alternative approach, namely administering an anti-Slamf1 monoclonal antibody to infected BALB/c mice once a week during the four weeks post infection with *T. cruzi*. We used a lower parasite inoculum than in *Slamf1* KO mice in order to increase the survival and to allow the action of the antibody. Based on analysis of parasite DNA (Figure 7), the number of amastigotes in the heart was significantly reduced in antibody treated mice as compared to mice that had received an isotype control. In addition IFNγ mRNA levels were also partially reduced in a similar fashion. As in the *Slamf1−/−* mouse the monoclonal antibody directed against Slamf1 did not affect the parasitemia in the blood (data not shown). Thus, the antibody experiments directly support the conclusions that are based upon the experiments obtained with *Slamf1−/−* mice.

## Discussion

The current studies led the concept that Slamf1 is required for replication of *T.cruzi* in macrophages and DCs, but not in other cells, which do not normally express the receptor, *e.g.* cardiomyocytes. Besides, in the absence of Slamf1, macrophages and DCs produce less myeloid cell specific factors that are key in influencing the host response to parasite and eventually the outcome of the infection. This explains why *T. cruzi* infected *Slamf1−/−* mice do not succumb to myocarditis induced by a lethal challenge with the highly virulent *T. cruzi* Y strain quite the opposite to BALB/c mice even with similar parasitemia levels. The later also indicates that parasitemia does not necessarily need to be related to cardiomyopathy, which is the leading cause of death upon *T.cruzi* infection in most instances [2], [9].

Our results also show that the systemic alterations previously reported associated to *T. cruzi* infection and suggested to play a role in pathology as impairment of thymocyte development [28], [32] or altered systemic cytokine production, among others [9], were not different in both infected *Slamf1−/−* mice and control *Slamf1+/+* mice, indicating that those major changes in systemic immune responses are not responsible for the reduced susceptibility of *Slamf1−/−* mice to *T. cruzi* infection. Rather, the altered local heart response to infection, with much lower *T. cruzi* amastigotes and altered immune mediators in infected *Slamf1^−/−^* mice, are the most likely cause for the survival of *Slamf1^−/−^* mice upon an acute infection by the parasite.

We favor an interpretation of our observations that in *Slamf1−/−* mice less *T. cruzi* parasites enter the heart. However, as circulating parasite levels are similar in *Slamf1−/−* mice and the *in vitro* susceptibility of cardiomyocytes to infection is not altered by Slamf1 deficiency, is likely that *T. cruzi* blood trypomastigotes are unable to penetrate the heart directly to infect the cardiomyocytes. On the other hand, *T. cruzi* replicate much less well in *Slamf1−/−* DC and macrophages than in the equivalent *wt* cells. Although Slamf1 is expressed on the surface of hematopoietic stem cells, a careful analysis of *Slamf1−/−* on two genetic backgrounds has not revealed any abnormalities in hematopoiesis included myeloid cells [23], [27]. It is therefore unlikely that a major defect in myeloid development and differentiation in *Slamf1−/−* mice has an effect on *T.cruzi* infection.

Thus, despite comparable infiltration of macrophages and DCs between infected *Slamf1^−/−^* mice *Slamf1−/−*, the number of infective amastigotes that are carried into the heart by infected migrating *Slamf1−/−* monocytes, DCs or macrophages will be greatly reduced and might be one of the contributing factors to the survival of *Slamf1−/−* mice to infection with the parasite. In addition, it is also possible that homing of infected monocytes or macrophages into the heart is affected by the absence of Slamf1. Together, our results suggest that DC and myeloid cells can act as a “Trojan horse” for *T. cruzi* infection into the heart.

An alternative interpretation is that the reduced amastigote number in the heart of Slamf1−/− mice is a result of a stronger response to *T. cruzi* that limits its replication. Collectively, our results argue against this, since immune cells infiltrate the heart of a *Slamf1−/−* mouse in a similar fashion as in a *BALB/c* mouse and the amounts of key cytokines produced in the heart are equal or lower than in the *wt* mouse. Thus, although IFNγ-producing CD8 T cells may enter the heart from the circulation to eliminate *T. cruzi* infected cells [33], in *Slamf1−/−* mice similar numbers of CD8 cells infiltrate the heart, but produce less IFNγ due to lower antigenic stimulus. Moreover, arginase I levels are lower in the heart of *Slamf1−/−* mice, and we have found that the levels of this enzyme presents in infiltrating myeloid suppressor cells correlate with higher susceptibility to infection [31]. This reduction may also contribute to explain the lower susceptibility of *Slamf1−/−* mice.

Parasitemia levels are similar in both mice strains. This fact suggests that circulating parasite levels are mostly due to replication of *T. cruzi* in other organs and cells others than myeloid cells. *T. cruzi* are known to replicate in many cell types, including muscle, epithelial and endothelial cells [9], [34]. Since Slamf1 is only expressed in myeloid cells, the replication of *T. cruzi* in non-hematopoietic cells is not likely to be impaired in *Slamf1−/−* mice and hence the blood-borne parasitemia is only slightly less in the mutant mice.

Previously, Slamf1 was found to be a requisite for the elimination of the *T. cruzi*-related intracellular protozoa (*Leishmania major*) by *B6* mice [23]. However, *Slamf1−/−* BALB/c mice respond to a *L. major* infection in an identical fashion as *wt* BALB/c animals. The role of Slamf1 in the response to the two related parasites is therefore different, as Slamf1 plays a detrimental role in *T. cruzi* infection of BALB/c mice. Consequently the mechanisms involved are likely to be different. In *Leishmania* infection the susceptibility of *Slamf1^−/−^* mice has been linked to a depressed NO production by macrophages, with a consequent inability to eliminate the parasite. NO is also required for *T. cruzi* killing [10] and we also found that *T. cruzi* infection in *Slamf1−/−* macrophages does not trigger iNOS, but this has no apparent impact for “in vitro” or “in vivo” replication. The reasons for this apparent discrepancy may lie in the fact that Slamf1 affects a different and earlier process in the infection of macrophages by *T. cruzi* than *L. major*, as the two parasites invade the cells by different mechanisms.

Besides, *Slamf1−/−* mice are also more susceptible to an attenuated strain of *S. tyhimurium* Sseb-e [27] contrary to *T. cruzi*. Although it might at face value appear paradoxical those contrasting effects one should keep in mind that in humans Slamf1 is one of the two receptors (probably the original receptor) for Measles virus [26], [27]. Therefore the virus and the parasite utilize an important receptor system to their advantage.

The diminished replication of *T. cruzi* in *Slamf1−/−* myeloid cells may explain, at least partially, the lower susceptibility of *Slamf1−/−* mice. Although, the mechanism by which Slamf1 reduce *T. cruzi* replication has not been addressed in this manuscript, previous experiments demonstrated that Slamf1 is involved in entering *E.coli* into phagosome, where it governs phago-lysosomal maturation and NADPH-oxidase (Nox2) activity [27]. This is caused by a reduction in of phosphatidyl-inositol 3-phosphate production, which is synthesized by the intracellular Class II PI3-kinase (PI3K) Vps34. As *T. cruzi* requires phagosome formation and PI3K (Vps34) activation to invade macrophages [17], [35], it is therefore likely that Slamf1 participates with other molecules/receptors in the entry of *T. cruzi* into the phagosome.

Moreover, a recent study shows that Nox2 inhibition ameliorates *T. cruzi*-induced myocarditis independently of parasitemia levels [36] as in *Slamf1−/−* mice. Thus, a reduced Nox-2 production together with a reduced replication in myeloid cells are the underlying mechanisms, which may explain the survival of *Slamf1^−/−^* mice to *T.cruzi* infection.

Interestingly, anti-Slamf1 treatment might affect the same processes. Consequently, Slamf1 is a key molecule in *T. cruzi* infectivity and represents an attractive novel therapeutic target for modulating *T. cruzi* infection and Chagas' disease.

## Materials and Methods

### Ethics statement

The animal research described in this manuscript complied with Spanish (Ley 32/2007) and European Union legislation (2010/63/UE). The protocols used were approved by the Animal Care Committee of the Centro de Biologia Molecular and Universidad Autonoma de Madrid.

### Parasites, mice and infections

*T. cruzi* Y strain epimastigotes were cultured in liver-infusion tryptose medium (LIT) supplemented with 10% FCS. Epimastigotes were differentiated into infective metacyclic trypomastigotes in GRACE medium (GIBCO BRL, Gran Island, NY) supplemented with 10% FCS for 10–12 days as described [17]. Blood trypomastigotes were maintained by weekly *i.p.* inoculations to BALB/c mice in our animal facilities. Six to 8-week-old *BALB/c* and *Slamf1^−/−^* mice [23], crossed 10 times with *BALB/c* mice as described, were maintained under pathogen-free conditions. Mice were infected *i.p* with 2×10^3^ typomastigotes of the Y strain and parasitemia was measured as described [37]. Animals were also infected *i.p* with 1×10^2^ typomastigotes and treated with the rat-anti-mouse Slamf1 antibody (9D1) or control rat antibody; 0.5 mg/mouse before the infection and then once a week.

### Cell cultures and infection

Neonatal, mouse primary cardiomyocyte cultures were obtained as described [30], [38]. More than 90% of cells were cardiomyocytes as detected by immunostaining with antibody to mAchR M2 as described [39]. After 24 h, the cultures were infected with *T. cruzi* as described. Spleen from infected or control mice were isolated as described [17]. Thymic cells were also obtained as described [40] and cells harvested and centrifuged three times in phosphate buffered saline containing 2% bovine serum albumin (Sigma) and 0.1% sodium azide. Later they were analyzed by flow cytometry using double immunofluorescence staining with phycoerythrin (PE)-anti-CD4 and fluorescein isothiocyanate (FITC)-anti-CD8α (Becton Dickinson).

Spleen cells were stained for flow citometry using monoclonal antibodies against CD45R/B220, CD11b, CD11c, CD4 and CD8 (BD Biosciences). All samples were acquired in a FACSCalibur cytometer (BD Biosciences) and analysed by using Flowjo 4.1 software (Tree Star, Inc).

Peritoneal cells from infected mice were collected with 0.34 M sucrose. Cells were then plated in complete RPMI with 5% FBS. After 4 h, non-adherent cells were removed by washing three times with warm PBS, and fresh complete RPMI was restored. Cells were analyzed by Giemsa staining under a light microscope. For *in vitro* infection, primary macrophages were isolated by peritoneal lavage of mice 4 days after a single intra-peritoneal injection of 10% thioglycolate solution (1 ml; Difco Laboratories). Cells (1.5×106/well) were allowed to adhere for 1 h in 12-well flat-bottomed plates in RPMI 0,5% FCS. Cells were co-cultured with *T. cruzi* metacyclic trypomastigotes (10 parasites/cell) for 4 h to allow binding and internalization and macrophages were washed with PBS four times to remove unbound parasites. Cells were analyzed 24 h and 48 h post infection for gene expression by RT-PCR or cytokine production by ELISA in the supernatants. IL-2, IL-12, IL-10, IFN-γ, TNF, IL-6, IL-17A and IL-4 in cell culture supernatants or in serum samples were evaluated using ELISA kits from R&D systems following manufacturer instructions.

Dendritic cells were obtained from bone marrow from the femur and tibia of mice was flushed of the hind limbs with ice cold PBS (phosphate-buffered saline), was centrifuged and resuspended into RPMI 1640 (GIBCO) 5% FBS (complete medium) supplemented with 20 ng/ml of recombinant murine GM-CSF (Peprotech) at 37°C, 5% CO2. Each three days 25 ml fresh medium was added with the same concentration of GM-CSF. At day 7 the medium was collected, centrifuged and the pellet resuspended in fresh medium. 1×10^6^ cells were plated in 6- well plates overnight in complete medium. Then, the cells were co-cultivated with *T. cruzi* trypomastigotes (10 parasites/cell) for 4 hours. After this time the cells were washed to remove the remaining parasites and they were incubated in fresh complete medium for the indicated time.

### Western blotting

Cells were lysed in NP-40 buffer (20 mM Tris- Hal pH 7.4, 1% Triton X-100, 150 mom Nail, 0.5% deoxyglycolate, 0.1% SDS and 10 mM NaF; the protease inhibitors aprotinin and leupeptin at 2 mg/ml, 1 mg/ml pepstatin and 1 mM PMSF; and 100 mM of the phosphatase inhibitor Na_3_VO_4_) for 30 min at 4°C, and supernatants were collected after centrifugation. The extracts (20 µg) were separated by SDS-PAGE (10% polyacrylamide) and subjected to Western blot with the appropriate antibodies for 1 h. Membrane was incubated with secondary antibody coupled to peroxidase and was revealed by Supersignal reagent (Pierce) and protein detected by autoradiography.

### Real time PCR for parasite DNA detection and mRNA analysis by quantitative RT-PCR

Parasite DNA was isolated from heart tissue after blood perfusion with the High Pure PCR Template preparation Kit (Roche) and PCR reactions were conducted with 100 ng of the DNA as described [31]. For *T. cruzi* detection, we followed the method described by Peron et al. [41]. Heart RNA was extracted with TRIzol reagent (Invitrogen).Quantitative RT-PCR analysis was done with High Capacity cDNA Archive Kit (Applied Biosystems) and amplification of different genes (*ArgI, inos2, B220, CD11c, CD4, CD8, CD68, Ifng, Tnf, il4, il10, il13, il12, ptgs2* and 18SrRNA) was performed in triplicate using TaqMan MGB probes and the TaqMan Universal PCR Master Mix (Applied Biosystems) on an ABI PRISM 7900 HT instrument (Applied Biosystems) as described [30]. Quantification of parasite DNA and gene expression by real-time PCR was calculated by the comparative threshold cycle (C*_T_*) method, normalized to the ribosomal 18S control and efficiency of the reverse transcription reaction (RQ =  2^−ΔΔC^ *_T_*). (Fold-change). Graphs were plotted as log RQ when indicated.

### Creatine Kinase (CK) assay

The activity of CK-MB isoenzyme, one of myocardial injury marker, was measured with commercial kits (EnzyChrom – ECPK-100, BioAssay Systems, USA) as described by the supplier. Incubation of serum samples with the substrate led to a net increase in NADPH concentration, directly proportional to the enzyme activity in the samples. The assay was adapted for reading in a microplate spectrophotometer (Microplate Nunclon Surface; FLUOstar Optima-BMG-Latch), to allow the study of small quantities of mouse serum according to the manufacturer's recommendation. The optical density at 340 nm was recorded at 10 min and again at 40 min.

### Histological analysis of heart

The hearts of mice infected or not were fixed in 10% neutral buffered paraformaldehyde, and embedded in paraffin. Longitudinal cuts of 5 µm thick were mounted on glass slides and stained with Haematoxylin-Eosin. The number of amastigote nests was estimated by observing 20 fields per preparation (each in triplicate) using the Lexica light microscope at a resolution of 630×. Hearts were also analyzed by confocal immunofluorescence as described [30]. Briefly, hearts were fixed in 4% paraformaldehyde in PBS solution, incubated in 30% sucrose solution, embedded in Tissue-Tek O.C.T. compound (Sakura) and frozen. Incubation with the following antibodies was done at 4°C: rat anti–mouseCD68-Alexa 594 (Serotec), goat anti- mouse CD11c-Alexa 555 (Santa Cruz), rat anti-mouse CD4-Alexa 647 (BD Pharmingen) and rat anti-mouse CD8-Alexa 594 (e-Bioscience). Images were obtained using an LSM510 Meta.

### Statistical analysis

For *in vivo* experiments, data reported are means ± SD from triplicate determination of a representative experiment out of at least three (n≤5). Results shown from *in vitro* experiments are representative of at least three experiments performed in duplicate. Significance was evaluated by Student's two-tailed *t*-test; all differences mentioned were significant compared to controls (p<0.05 or p<0.01).

## Supporting Information

Figure S1 **Spleen cell populations in infected mice.** Splenocytes were isolated from thymus from control NI or *T. cruzi* infected BALB/c or *Slamf1^−/−^* mice at 14 and 21 dpi. The percentage of major leukocyte subpopulations in the spleen was assessed by flow cytometry. Results are expressed as the mean values (±SD) for triplicates and from 5 different mice in each group.
(TIF)

Figure S2 **Heart cytokine production by** ***T. cruzi*** **infected mice.** Cytokine mRNA production in the heart of *T. cruzi* infected mice was evaluated by QC-PCR as described in Methods. Total RNA was isolated in heart tissue obtained from BALB/c and *Slamf1^−/−^* mice at different days post infection (dpi), and quantitative reverse-transcriptase polymerase chain reaction was performed as described in Materials and Methods. Results are expressed as the logarithm of relative quantity (RQ) calculated from comparative threshold cycle values, as described in Material and Methods.
(TIF)

Figure S3 **Cytokine production in the serum of infected mice.** The levels of different cytokines (IFN- γ, TNF, IL-2, IL-4, IL-6, IL-10, IL-12 and IL-17A) were quantified in blood of control and infected mice by flow cytometry following the instructions indicated by the supplier (Cytometric Bead Array-Becton Dickinson). Results are expressed as the mean values (±SD) for triplicates from 3 different mice. A representative experiment of the 3 performed is shown. (*) Statistically significant differences between *Slamf1^−/−^* mice and BALB/c (p>0.05).
(TIF)
