# Supplementary figures and images for: The Receptor Slamf1 on the Surface of Myeloid Lineage Cells Controls Susceptibility to Infection by Trypanosoma cruzi
Source: PLoS Pathog. 2012 Jul 12;8(7):e1002799. doi: 10.1371/journal.ppat.1002799 (PMC3395606; doi:10.1371/journal.ppat.1002799)

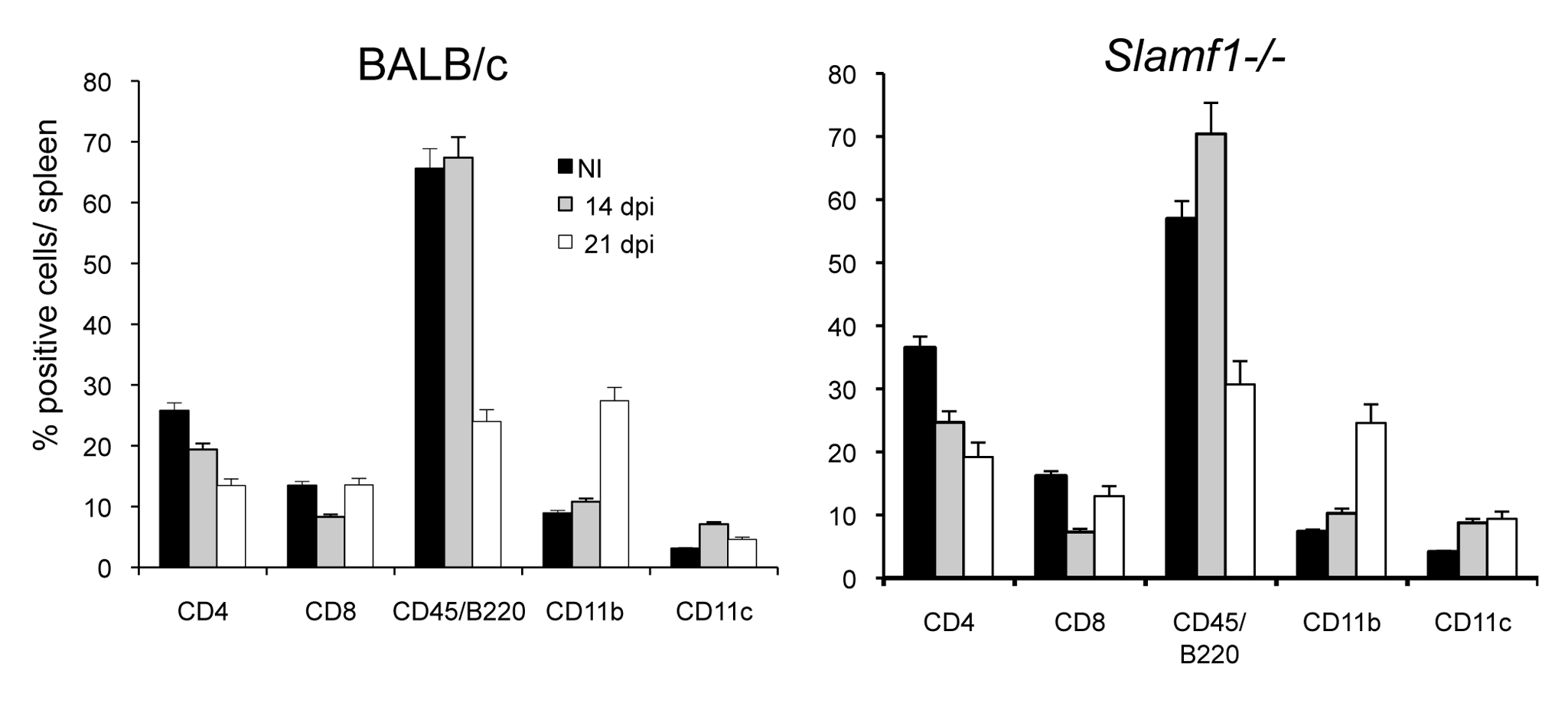

Supplement: Figure S1 — Spleen cell populations in infected mice. Splenocytes were isolated from thymus from control NI or T. cruzi infected BALB/c or Slamf1−/− mice at 14 and 21 dpi. The percentage of major leukocyte subpopulations in the spleen was assessed by flow cytometry. Results are expressed as the mean values (±SD) for triplicates and from 5 different mice in each group. (TIF) [file ppat.1002799.s001.tif]

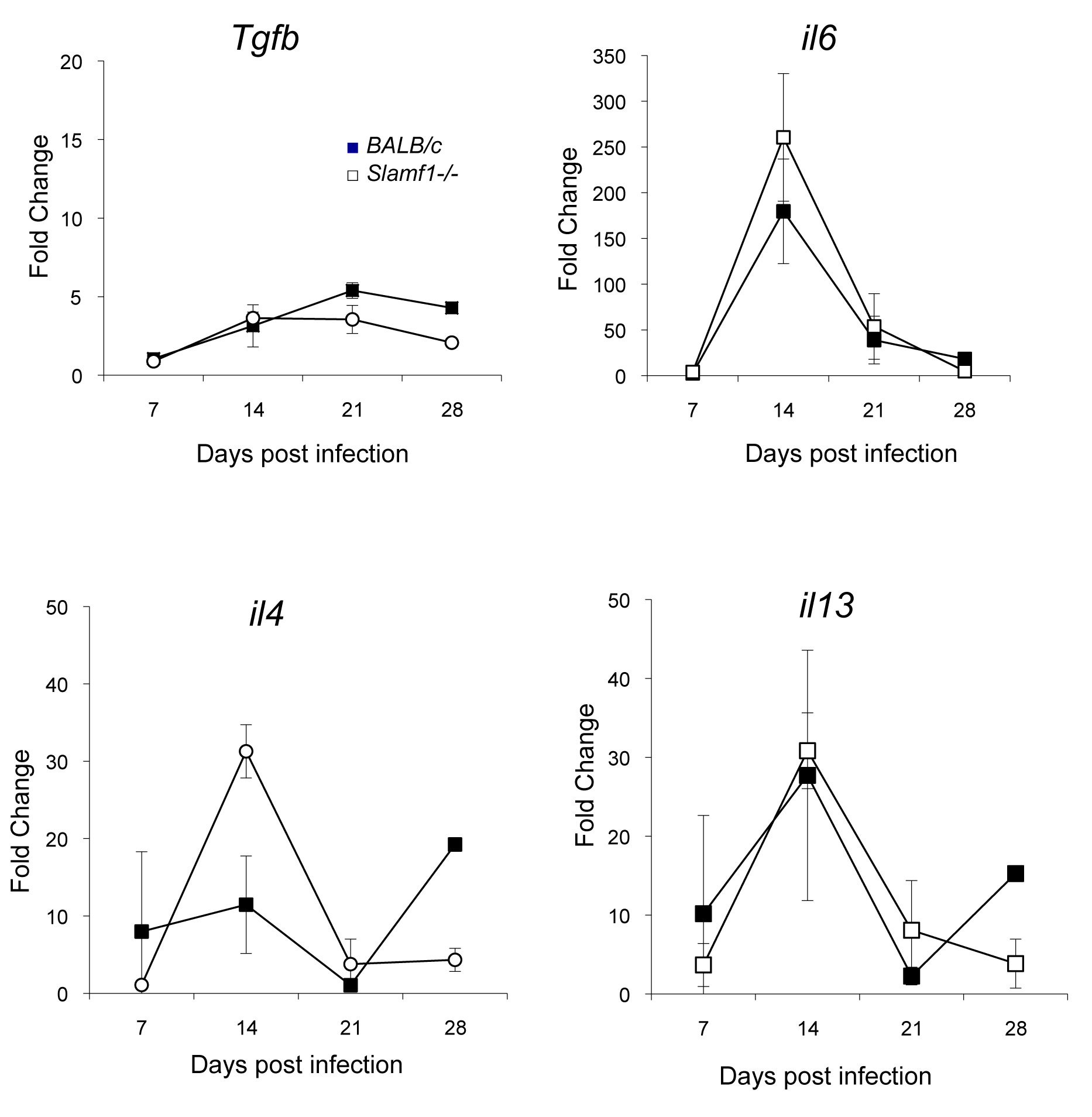

Supplement: Figure S2 — Heart cytokine production by T. cruzi infected mice. Cytokine mRNA production in the heart of T. cruzi infected mice was evaluated by QC-PCR as described in Methods. Total RNA was isolated in heart tissue obtained from BALB/c and Slamf1−/− mice at different days post infection (dpi), and quantitative reverse-transcriptase polymerase chain reaction was performed as described in Materials and Methods. Results are expressed as the logarithm of relative quantity (RQ) calculated from comparative threshold cycle values, as described in Material and Methods. (TIF) [file ppat.1002799.s002.tif]

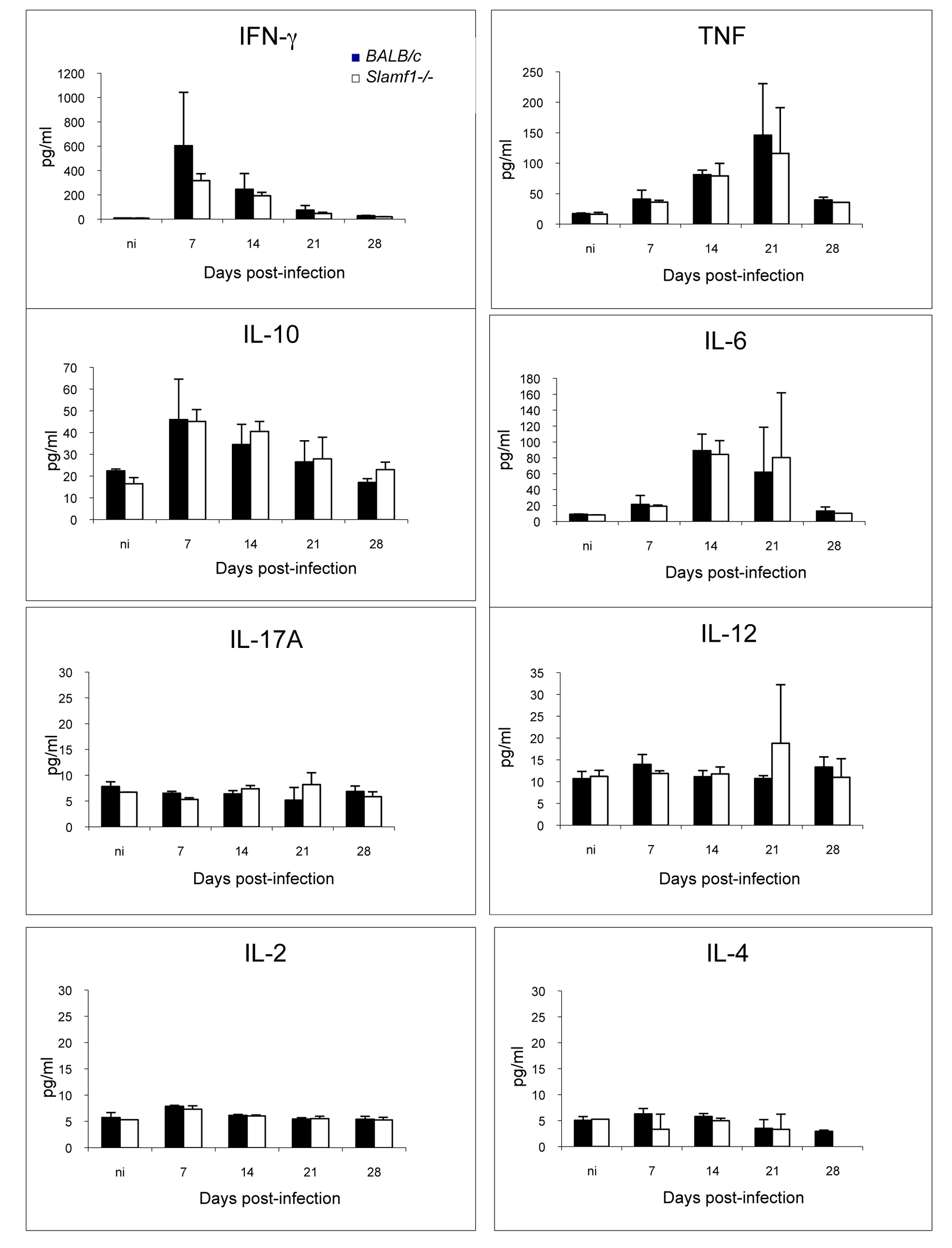

Supplement: Figure S3 — Cytokine production in the serum of infected mice. The levels of different cytokines (IFN- γ, TNF, IL-2, IL-4, IL-6, IL-10, IL-12 and IL-17A) were quantified in blood of control and infected mice by flow cytometry following the instructions indicated by the supplier (Cytometric Bead Array-Becton Dickinson). Results are expressed as the mean values (±SD) for triplicates from 3 different mice. A representative experiment of the 3 performed is shown. (*) Statistically significant differences between Slamf1−/− mice and BALB/c (p>0.05). (TIF) [file ppat.1002799.s003.tif]
